# Composition of the ileum microbiota is a mediator between the host genome and phosphorus utilization and other efficiency traits in Japanese quail (*Coturnix japonica*)

**DOI:** 10.1186/s12711-022-00697-8

**Published:** 2022-03-08

**Authors:** Valentin Haas, Solveig Vollmar, Siegfried Preuß, Markus Rodehutscord, Amélia Camarinha-Silva, Jörn Bennewitz

**Affiliations:** Institute of Animal Science, University of Hohenheim, 70599 Stuttgart, Germany

## Abstract

**Background:**

Phosphorus is an essential nutrient in all living organisms and, currently, it is the focus of much attention due to its global scarcity, the environmental impact of phosphorus from excreta, and its low digestibility due to its storage in the form of phytates in plants. In poultry, phosphorus utilization is influenced by composition of the ileum microbiota and host genetics. In our study, we analyzed the impact of host genetics on composition of the ileum microbiota and the relationship of the relative abundance of ileal bacterial genera with phosphorus utilization and related quantitative traits in Japanese quail. An F2 cross of 758 quails was genotyped with 4k genome-wide single nucleotide polymorphisms (SNPs) and composition of the ileum microbiota was characterized using target amplicon sequencing. Heritabilities of the relative abundance of bacterial genera were estimated and quantitative trait locus (QTL) linkage mapping for the host was conducted for the heritable genera. Phenotypic and genetic correlations and recursive relationships between bacterial genera and quantitative traits were estimated using structural equation models. A genomic best linear unbiased prediction (GBLUP) and microbial (M)BLUP hologenomic selection approach was applied to assess the feasibility of breeding for improved phosphorus utilization based on the host genome and the heritable part of composition of the ileum microbiota.

**Results:**

Among the 59 bacterial genera examined, 24 showed a significant heritability (nominal p ≤ 0.05), ranging from 0.04 to 0.17. For these genera, six genome-wide significant QTL were mapped. Significant recursive effects were found, which support the indirect host genetic effects on the host’s quantitative traits via microbiota composition in the ileum of quail. Cross-validated microbial and genomic prediction accuracies confirmed the strong impact of microbial composition and host genetics on the host’s quantitative traits, as the GBLUP accuracies based on the heritable microbiota-mediated components of the traits were similar to the accuracies of conventional GBLUP based on genome-wide SNPs.

**Conclusions:**

Our results revealed a significant effect of host genetics on composition of the ileal microbiota and confirmed that host genetics and composition of the ileum microbiota have an impact on the host’s quantitative traits. This offers the possibility to breed for improved phosphorus utilization based on the host genome and the heritable part of composition of the ileum microbiota.

**Supplementary Information:**

The online version contains supplementary material available at 10.1186/s12711-022-00697-8.

## Background

The poultry industry is a fast-growing sector of the global food supply. For economic and environmental impact reasons, feed efficiency and nutrient efficiency have received considerable attention in poultry research. Phosphorus (P) is an essential nutrient with finite global mineral resources and an enormous environmental impact due to its excretion in animal faeces [1–3]. In plant seeds, P is primarily stored in the form of phytic acid (myo-inositol hexaphosphate, InsP_6_) [4]. Better utilization of P from feed components is a desirable goal, which requires the action of phytase and other phosphatase enzymes that catalyze the stepwise cleavage of P from InsP_6_ in the digestive tract [5, 6]. Poultry are known to have comparably low endogenous phytase activity. Nevertheless, native InsP degradation in the gastrointestinal tract (GIT) can occur by the action of phytases and phosphatases that originate from the endogenous mucosa of the GIT from some vegetable feed components, or from the gut microbiota (reviewed in [7]).

It is well known that microbiota composition in the GIT of livestock is influenced by environmental factors, such as diet or housing conditions. However, numerous literature results indicate that host genetics has also an effect on GIT microbial colonization; and significant heritabilities have been reported for the relative abundance of bacterial genera in the cow rumen [8, 9], for bacterial genera and operational taxonomic units (OTU) level in the pig colon [10, 11], and for cecal and fecal microbial species in chickens [12–14].

A previous study on Japanese quails under standardized feeding and housing conditions showed that P utilization (PU) varied substantially between birds and had a heritability of 0.14 [15]. Borda-Molina et al. [16] detected differences in the relative abundance of different microbial genera between quails from the same population with high- versus low-PU. In a subsequent study, we confirmed that PU and related traits are strongly influenced by the hosts’ composition of the ileal microbiota and we estimated a significant microbiability with an order of magnitude similar to heritability [17]. We assessed the microbial architecture of PU and related host traits by applying microbiome-wide association analysis (MWAS) and found that they were polymicrobial, with many trait-associated bacterial genera, but none of the genera had an exceptionally large effect [17]. Subsequently, we used the same dataset to map quantitative trait loci (QTL) for PU in the quail genome. The quails were genotyped with 4k single nucleotide polymorphisms (SNPs) and several significant QTL were identified [18].

In MWAS, it is assumed that the microbiota composition is the cause of the variation in host’s quantitative traits [17], but this might not be true because microbiota composition and quantitative traits could be influenced by the same host QTL and thus may be correlated via a common set of host QTL. Structural equation models (SEM) [19] are multivariate mixed model equations that account for recursive relationships between traits and allow separation of direct and indirect genetic effects that are responsible for the genetic relationship between traits. If a recursive relationship exists, QTL that directly affect one trait may indirectly affect the second trait via the recursive relationship between the traits. Saborío-Montero et al. [8] used SEM to identify significant polymicrobial recursive interactions between rumen microbiota and methane emissions in cattle. Tiezzi et al. [20] also confirmed recursive effects of fecal microbiota composition on fat deposition in pigs, using SEM.

In Weishaar et al. [21], a genome-based selection index to improve a quantitative trait in the host was developed that considered the hologenome, i.e., both the host genome and microbial metagenome of microbiota composition in the GIT. The selection index included estimated breeding values for the direct effect of the host genome on the trait and for the indirect effect mediated by microbiota composition. The core aspect of the method is a reference population with trait-recorded animals that have been genotyped for a SNP‐chip and characterized for microbiota composition. A microbial mixed model was used to estimate the effect of the animal’s microbiota on the trait. Subsequently a genomic mixed linear model was applied to predict the SNP effects for the estimated animal microbiota effect. Weishaar et al. [21] successfully applied this model to a small pig dataset on feed efficiency.

Using the same quail dataset as in our previous studies [17, 18], the aim of the current study was to analyze the impact of host genetics on composition of the ileum microbiota and the relationship of ileal bacterial genera with PU and related traits, i.e. body weight gain (BWG), feed intake (FI) and feed per gain (F:G). For this purpose, we estimated heritabilities of the relative abundance of bacterial genera, and the correlations and recursive relationships between the relative abundance of the bacterial genera and these four traits, and performed QTL linkage mapping for the relative abundance of bacterial genera. Subsequently, the hologenomic selection approach developed by Weishaar et al. [21] was applied to assess the feasibility of breeding for improved PU based on the host genome and the heritable part of the composition of the ileum microbiota.

## Methods

### Experimental design

Details of the experimental design are in Beck et al. [15] and, thus, only the most relevant aspects are presented in the following. This animal experiment was performed according to the requirements of the German Animal Welfare Legislation and was approved by the Animal Welfare Commissioner of the University of Hohenheim (approval number S371/13TE). An F2 population of Japanese quail (*Coturnix japonica*) was established based on two divergent lines selected for social reinstatement behavior [22]. Twelve males and 12 females from each founder line were mated to generate the F1 generation. Seventeen roosters and 34 hens from the F1 generation were randomly selected and mated (one male with two females), resulting in 920 F2 individuals. These F2 birds were phenotyped between 10 and 15 days of age, while the birds were provided with a corn-soybean meal-based diet without mineral P or phytase supplements. A diet with an overall low P content was chosen to evaluate the PU potential of the quails.

### Sample collection, SNP genotyping, and characterization of the ileum microbiota

The focal trait of this experiment was PU, which was calculated based on total P intake and P excretion, as well as based on FI during the experimental period. Quail BWG was quantified as the difference in body weight between days 10 and 15. The F:G ratio was computed as the FI during this 5-day period divided by the BWG. The quails were slaughtered at 15 days of age to collect ileum samples for further analysis. The birds were incubated and slaughtered on 12 different days, which were treated as test days in the statistical analysis. Estimates of the phenotypic and genetic correlations between the four recorded traits are in Beck et al. [15] and Künzel et al. [23].

DNA preparation, 4k SNP genotyping, and construction of a genome-wide linkage map are described in detail by Vollmar et al. [18]. In brief, all birds were genotyped for 5388 SNPs and the following criteria were applied to filter the genotypes: SNPs with one or more conflicting genotypes between parent and offspring, a minor allele frequency (MAF) ≤ 0.03, a SNP call frequency ≤ 0.9, and a cluster separation ≤ 0.4 were removed. We also excluded SNPs on the sex chromosomes Z and W. Finally, 3986 SNPs remained for further analysis.

Analyses of the composition of the ileum microbiota were performed by target amplicon sequencing, as described in Borda-Molina et al. [16]. Sequences were clustered into OTU at > 97% similarity. In total, 1188 OTU with an average relative abundance higher than 0.0001% and a sequence length greater than 250 bp were used in further analyses. Representative sequences were manually identified with the seqmatch function of the RDP database [24]. The output taxonomy table followed the confidence threshold cut-off value for each taxonomic level as defined by Yarza et al. [25]: genus (94.5%), family (86.5%), order (82.0%), class (78.5%) and phylum (75.0%) [25]. Due to the use of a strict quality filter on the sequences, several samples were excluded. The final dataset included data on 758 quails with SNP genotypes, microbiota composition characteristics, and trait records (PU, FI, BWG, and F:G).

### Statistical analyses

#### Transformation of microbial data

We used two microbial classifications for the statistical analyses, i.e., microbial genus and OTU. The latter was used to build the microbial relationship matrix ${\mathbf{M}}$ (see below). Genera data were filtered for a minimum of 0.01% of the average relative abundance of each genus. This filtering step reduced the number of genera from 200 to 59. Because the distribution of the relative abundance of each microbial genus deviated remarkably from a Gaussian distribution, we applied a Box–Cox transformation with a specific lambda for each genus. The lambda was determined by a grid search to maximize the likelihood function of a normal distribution, following Box and Cox [26]:

$$f\left( {\mathbf{y}} \right) = \left\{ {\begin{array}{*{20}ll} {\frac{{{\mathbf{y}}\lambda - 1}}{\lambda } \left( {\lambda \ne 0} \right)} \\ {log\,{\mathbf{y}} (\lambda = 0)} \\ \end{array} } \right.,$$

where ${\mathbf{y}}$ is a vector of the relative abundances of each microbial genus to be transformed, and $\lambda$ is the transformation parameter determined for each genus, which ranged from − 2 to 0.505.

#### Mixed linear models for microbial composition

The following statistical analyses using a mixed linear model were performed in R Studio (Version 3.5.3) [27], ASReml R (Version 3.0) [28], and ASReml 4.1 [29]:

1 $${\mathbf{y}} = \mu {\mathbf{1}} + {\mathbf{Z}}_{{{\mathbf{td}}}} {\mathbf{td}} + {\mathbf{Z}}_{{\mathbf{a}}} {\mathbf{a}} + {\mathbf{e}},$$

where ${\mathbf{y}}$ is a vector of the transformed relative abundances of each genus, $\mu$ is the trait mean, and ${\mathbf{1}}$ is a vector of 1s; ${\mathbf{td}}$ is a vector of the random test day effects, assumed to follow a normal distribution ${\mathbf{td}} \sim \user2{ }N\left( {0, {\mathbf{I}}\sigma_{td}^{2} } \right)$, where $\sigma_{td}^{2}$ is the variance, ${\mathbf{I}}$ is the identity matrix, and ${\mathbf{Z}}_{{{\mathbf{td}}}}$ is the design matrix; ${\mathbf{a}}$ is a vector of the random animal effects, assumed to follow a normal distribution ${\mathbf{a}} \sim N\left( {0,{\mathbf{A}}\sigma_{a}^{2} } \right)$, where ${\mathbf{A}}$ is the pedigree-based relationship matrix and $\sigma_{a}^{2}$ the additive genetic variance, and ${\mathbf{Z}}_{{\mathbf{a}}}$ is the design matrix. We chose to use pedigree instead of SNP genotypes here, because of the limited number of SNPs in the study. Finally, ${\mathbf{e}}$ is a vector of random residuals, assumed to follow a normal distribution ${\mathbf{e}}\user2{ }\sim \user2{ }N\left( {0,{\mathbf{I}}\sigma_{e}^{2} } \right)$, where $\sigma_{e}^{2}$ is the variance.

Using this mixed linear model, heritability ($h_{y}^{2}$) of each microbial genus was estimated as $h_{y}^{2} = \frac{{\sigma_{a}^{2} }}{{\sigma_{p}^{2} }}$, with $\sigma_{p}^{2} = \sigma_{a}^{2} + \sigma_{td}^{2} + \sigma_{e}^{2}$. Significance of the heritabilities was tested by conducting a likelihood ratio test on the random animal effects. The test statistic was computed as $D = 2\left[ {log\left( {L_{2} } \right) - log\left( {L_{1} } \right)} \right]$, where $L_{2}$ is the likelihood of the full Model (1) and $L_{1}$ that of Model (1) without random animal effects, and is distributed as a chi-square with one degree of freedom under the null hypothesis of zero heritability. All microbial genus heritabilities with a nominal p value ≤ 0.05 were used for further analyses. To estimate the number of false positives among the significant heritabilities, we calculated the false discovery rate (FDR) q value [30], assuming 59 comparisons. The FDR q value of the significant heritability with the largest p value provided an estimate of the proportion of false positives among the significant heritabilities of the microbial genera.

#### Bivariate analyses of microbial composition with host traits using structural equation models

We estimated the phenotypic correlations as Pearson correlations between each significant heritable genus (p ≤ 0.05) and each of the four host traits (PU, FI, BWG, and F:G) using the function cor.test() in R [27]. Subsequently, bivariate SEM were applied to trait-genus combinations with a significant phenotypic correlation (p ≤ 0.05) in order to estimate phenotypic ($r_{p}$) and genetic correlations ($r_{g}$) and to reveal the biological link between heritable microbiota and host traits. Of the four host traits, only FI is assumed to affect microbiota compositions, e.g. by the rate of food passage in the gastrointestinal tract. However, for the sake of simplicity, a recursive relationship between the heritable microbial genera and each of the four traits was assumed. The following bivariate recursive mixed linear model was applied, using the notation of Rosa et al. [31]:

2 $${\mathbf{y}} = \left( {{{\varvec{\Lambda}}} \otimes {\mathbf{I}}_{{\mathbf{n}}} } \right){\mathbf{y}}\user2{ } + {\mathbf{Z}}_{{{\mathbf{td}}}} {\mathbf{td}} + {\mathbf{Z}}_{{\mathbf{a}}} {\mathbf{a}} + {\mathbf{e}},$$

where ${\mathbf{y}}$ is the vector of the phenotypic records of the two analyzed traits for the $n$ individuals in the identity matrix ${\mathbf{I}}_{{\mathbf{n}}}$. The off-diagonal 2 × 2 matrix ${{\varvec{\Lambda}}}$ contains the structural coefficients $\lambda_{i,j}$, which express the rate of change of trait $i$ (PU, FI, BWG or F:G) as a result of the recursive influence of trait $j$, i.e., the relative abundance of the microbial genera in the ileum:

$${{\varvec{\Lambda}}} = \left[ {\begin{array}{*{20}c} 0 & 0 \\ {\lambda_{i,j} } & 0 \\ \end{array} } \right].$$

The remaining terms are as defined in Model (1). The joint distribution of ${\mathbf{td}}$, ${\mathbf{a}}$, and ${\mathbf{e}}$ was as follows:

3 $$\left[ {\begin{array}{*{20}c} {{\mathbf{td}}} \\ {\mathbf{a}} \\ {\mathbf{e}} \\ \end{array} } \right]\sim N\left\{ { \left[ {\begin{array}{*{20}c} 0 \\ 0 \\ 0 \\ \end{array} } \right], \;\left[ {\begin{array}{*{20}c} {{\mathbf{T}} \otimes {\mathbf{I}}_{{\mathbf{m}}} } & 0 & 0 \\ 0 & {{\mathbf{G}} \otimes {\mathbf{A}}} & 0 \\ 0 & 0 & {{\mathbf{R}} \otimes {\mathbf{I}}_{{\mathbf{n}}} } \\ \end{array} } \right]} \right\},$$

where ${\mathbf{T}}$, ${\mathbf{G}}$, and ${\mathbf{R}}$ are the test day, additive-genetic, and residual variance–covariance matrices of the system of equations [19]. Identity matrices ${\mathbf{I}}_{{\mathbf{m}}}$ and ${\mathbf{I}}_{{\mathbf{n}}}$ have dimensions equal to the number of test days ($m$) and the number of individuals ($n$), respectively. Matrix ${\mathbf{A}}$ is the numerator relationship matrix, and $\otimes$ is the Kronecker product. In Model (3), we assumed a diagonal residual covariance matrix to ensure the identifiability [31]. The following transformations were applied to obtain genetic parameters that correspond to those of mixed linear models without recursive effects [19, 31, 32]:

4 $$\begin{aligned} {\mathbf{T}}^{*} & = \left( {{\mathbf{I}} - {{\varvec{\Lambda}}}} \right)^{ - 1} {\mathbf{T}}\left( {{\mathbf{I}} - {{\varvec{\Lambda}}}} \right)^{\prime - 1} \\ {\mathbf{G}}^{*} & = \left( {{\mathbf{I}} - {{\varvec{\Lambda}}}} \right)^{ - 1} {\mathbf{G}}\left( {{\mathbf{I}} - {{\varvec{\Lambda}}}} \right)^{\prime - 1} \\ {\mathbf{R}}^{*} & = \left( {{\mathbf{I}} - {{\varvec{\Lambda}}}} \right)^{ - 1} {\mathbf{R}}\left( {{\mathbf{I}} - {{\varvec{\Lambda}}}} \right)^{\prime - 1} \\ {\mathbf{P}}^{*} & = {\mathbf{T}}^{*} + {\mathbf{G}}^{*} + {\mathbf{R}}^{*} \\ \end{aligned}$$

Phenotypic and genetic correlations were estimated based on these matrices (${\mathbf{T}}^{*} ,\user2{ }{\mathbf{G}}^{*} ,\user2{ }{\mathbf{R}}^{*}$) using standard notations. Standard errors were estimated using the method described by Beck et al. [15]. Note that it would be possible to estimate genetic parameters from the ${\mathbf{T}}$, ${\mathbf{G}}$ and ${\mathbf{R}}$ matrices, which can be interpreted as ‘system parameters’ [19] that control the ‘system’ of the traits. Comparison of estimates of genetic parameters based on ${\mathbf{G}}$ versus ${\mathbf{G}}^{*}$ would also shed light on the nature of the genetic correlation, i.e. the extent to which it is driven by pleiotropy or by indirect effects. However, since this would result in many additional parameters with large standard errors due to the limited sample size, estimates based on the ${\mathbf{T}}$, ${\mathbf{G}}$ and ${\mathbf{R}}$ matrices are not shown.

#### QTL linkage analyses of microbial genera

For QTL mapping, we used the R package R/qtl2 [33], which was originally set up for inbred crosses. Because the founders in our study were not inbred, this assumption was not fulfilled. Therefore, we calculated the QTL genotype probabilities for each F2 individual and each chromosomal position using the R package MAPfastR [34], which was developed for outbred line crosses, which were then transferred to R/qtl2. Genome scans were performed using regression of the phenotypes on two QTL genotype probability***-***derived regression variables, representing the QTL additive and dominant effects [33]. The software did not allow the inclusion of random nuisance effects, other than a residual, or classification effects. Therefore, the effects of test days were included as dummy covariates in the model. The resulting logarithm of the odds (LOD) scores per cM were used as test statistics. To address the problem of multiple testing, a permutation test (10,000 permutations) was applied to derive 5 and 10% genome-wide significance thresholds for each microbial genus. Support intervals (SI) for QTL position were determined by using the 1.5 LOD drop***-***off method, which corresponds approximately to a 95% confidence interval [35].

Within the SI for each identified QTL, all markers were evaluated for trait association using the single-marker association mapping approach implemented in the software package GCTA [36]. The model regressed the phenotypes on the number of copies of the 1-allele at the SNP (i.e. 0, 1, or 2 copies) and included test days as dummy covariates and the random animal genetic effect with a SNP-derived covariance matrix, as implemented in the software using the LOCO option. No correction for multiple testing was performed during the association analysis within the SI because the number of SNPs within a SI was usually small, reducing the problem of multiple testing in genome-wide association analysis.

#### Genomic and microbial trait predictions

To evaluate the hologenomic selection index proposed by Weishaar et al. [21], we first applied a microbial linear mixed model to each quantitative trait as follows [17]:

5 $${\mathbf{y}} = \mu{\mathbf{1}} + {\mathbf{Z}}_{{{\mathbf{td}}}} {\mathbf{td}} + {\mathbf{Ik}} + {\mathbf{e}},$$

where ${\mathbf{y}}$ is the vector of observations of one of the four performance traits (PU, FI, BWG, or F:G) for $n$ animals and ${\mathbf{k}}$ is the vector of the random microbiota effects of all animals, assumed to be distributed as ${\mathbf{k}} \sim \user2{ }N\left( {0, {\mathbf{M}}\sigma_{k}^{2} } \right)$, where $\sigma_{k}^{2}$ is the microbial variance and ${\mathbf{M}}$ is the microbial relationship matrix calculated as described in Camarinha-Silva et al. [10]. The remaining terms are as defined in Model (1).

Estimates of the microbial animal effects ${\hat{\mathbf{k}}}$ for each trait from Model (5) were then used as observations in the following genomic prediction model, as proposed by Weishaar et al. [21]:

6 $${\hat{\mathbf{k}}} = \mu {\mathbf{1}} + {\mathbf{Im}} + {\mathbf{e}},$$

where $\mu$ is the overall mean, ${\mathbf{m}}$ is the vector of random animal genetic effects, assumed distributed ${\mathbf{m}}\user2{ }\sim \user2{ }N\left( {0, {\mathbf{G}}\sigma_{m}^{2} } \right)$, where ${\mathbf{G}}$ is the genomic covariance matrix, estimated using the 4k SNP genotypes following method 1 of VanRaden [37] and $\sigma_{m}^{2}$ is the genomic variance of the estimated microbiota effects; and ${\mathbf{e}}$ is the vector of residuals with variance $\sigma_{e}^{2}$. Heritability of the microbiota-mediated trait ${\hat{\mathbf{k}}}$ was calculated as $h_{k}^{2} = \frac{{\sigma_{m}^{2} }}{{\sigma_{m}^{2} + \sigma_{e}^{2} }}.$ Significance tests for estimates of heritability were performed by likelihood ratio tests.

Three types of predictions were performed and evaluated using cross-validation, two genomic predictions and on microbial prediction. Model (1) was used to obtain genomic best linear unbiased predictions (GBLUP), but with the ${\mathbf{A}}$ matrix replaced by the ${\mathbf{G}}$ matrix. Model (5) was used to obtain microbial (M)BLUP [10]. For GBLUP of the microbiota-mediated part of the trait, Model (5) was used to obtain estimates of the random microbiota effects of the animals for each of the four traits, which were subsequently used as observations in Model (6).

Microbial and genomic predictions were evaluated using cross-validation with 500 repetitions, with variance components fixed at their estimated values. For each repetition, a reference population of 80% of the animals was randomly selected to estimate the effects of OTU and/or SNPs. The remaining 20% of animals were used as the validation population for prediction of animal effects. The average Pearson correlation between the predicted animal effects and the observed animal phenotypes across replications were used as the accuracy of prediction, with confidence intervals calculated from the 2.5 and 97.5% quantiles of the 500 correlations.

## Results

### Heritabilities, correlations, and structural coefficients

Among the 59 bacterial genera examined, 24 had a significant estimate of heritability (p ≤ 0.05) (Table 1), with estimates ranging from 0.04 to 0.17. The highest estimates were for *Clostridium *sensu stricto, *Lactobacillus* and *Bifidobacterium*, at 0.17, 0.12 and 0.10, respectively. All but one of the heritable genera belonged to the Firmicutes and Actinobacteria phyla. The average relative abundance of the heritable genera ranged from 0.01 to 24.33%.

**Table 1:** Bacterial genera with significant heritability estimates (p ≤ 0.05)

Phylum	Genus	Average relative abundance (%)	Heritability	SE	p value	False discovery rate
Actinobacteria	*Bifidobacterium*	0.48	0.10	0.05	< 0.001	< 0.001
Firmicutes	*Clostridium *sensu stricto	14.11	0.17	0.07	< 0.001	< 0.001
Firmicutes	*Lactobacillus*	24.33	0.12	0.05	< 0.001	< 0.001
Firmicutes	*Macrococcus*	0.23	0.06	0.03	< 0.001	0.002
Proteobacteria	*Escherichia/Shigella*	14.17	0.09	0.05	0.001	0.008
Actinobacteria	*Cutibacterium*	0.06	0.08	0.04	0.002	0.019
Firmicutes	*Aerococcus*	0.47	0.08	0.04	0.003	0.025
Firmicutes	*Bacillus*	0.08	0.06	0.03	0.006	0.042
Firmicutes	*Staphylococcus*	0.31	0.05	0.03	0.006	0.042
Firmicutes	*Tyzzerella*	0.08	0.07	0.04	0.007	0.042
Firmicutes	Unc*. Lachnospiraceae*	0.38	0.06	0.03	0.010	0.051
Firmicutes	*Enterococcus*	3.75	0.06	0.04	0.011	0.055
Actinobacteria	*Corynebacterium*	0.47	0.06	0.04	0.012	0.055
Actinobacteria	*Curtobacterium*	0.01	0.06	0.04	0.014	0.058
Firmicutes	*Streptococcus*	8.25	0.08	0.05	0.022	0.088
Actinobacteria	*Microbacterium*	0.02	0.05	0.03	0.026	0.094
Firmicutes	*Sellimonas*	0.03	0.05	0.03	0.028	0.094
Firmicutes	*Leuconostoc*	0.12	0.04	0.03	0.029	0.094
Firmicutes	*Ruminococcus 2*	0.28	0.05	0.03	0.030	0.094
Firmicutes	*Anaerofilum*	0.02	0.04	0.03	0.040	0.108
Firmicutes	*Anaerostipes*	0.06	0.04	0.03	0.040	0.108
Firmicutes	*Lactococcus*	0.14	0.04	0.03	0.040	0.108
Actinobacteria	*Corynebacterium*	0.15	0.05	0.04	0.043	0.109
Firmicutes	*Subdoligranulum*	0.07	0.05	0.03	0.049	0.121

*SE* standard errors

Table 2 shows estimates of genetic $(r_{g} )$ and phenotypic $\left( {r_{p} } \right)$ correlations and of the structural coefficients $\lambda_{PU,Genus}$, $\lambda_{FI,Genus}$, $\lambda_{BWG,Genus}$ and $\lambda_{F:G,Genus}$ between the microbial genera and each of the four traits, for all genera with a significant Pearson correlation (p ≤ 0.05) and a significant structural coefficient (p ≤ 0.05) between the genera and each of the four traits. Genetic correlations estimates between the considered genera and the four phenotypes had large standard errors due to the limited number of animals and ranged from − 0.19 (*Enterococcus*) to 0.52 (*Lactococcus*) for PU, from − 0.03 (*Microbacterium*) to 0.47 (*Leuconostoc*) for FI, from − 0.69 (*Enterococcus*) to 0.48 (*Lactococcus*) for BWG, and from − 0.38 (*Lactococcus*) to 0.54 (*Enterococcus*) for F:G. Ranges for estimates of phenotypic correlations were narrower, from − 0.09 (*Streptococcus*) to 0.14 (*Bacillus*) for PU, from 0.17 (*Microbacterium*) to 0.31 (*Leuconostoc*) for FI, from − 0.16 (*Macrococcus*) to 0.25 (*Leuconostoc*) for BWG, and from − 0.38 (*Lactococcus*) to 0.17 (*Enterococcus*) for F:G. Estimates of the standardized recursive effects, $\lambda_{i,Genus}$, ranged from − 0.039 ($\lambda_{PU,Enterococcus}$) to 0.081 ($\lambda_{PU,Bacillus} )$ for PU, from 0.028 ($\lambda_{FI,Microbacterium}$) to 0.095 ($\lambda_{FI,Bacillus}$) for FI, from − 0.056 ($\lambda_{BWG,Streptococcus}$) to 0.102 ($\lambda_{BWG,Bacillus}$) for BWG, and from − 0.064 ($\lambda_{F:G,Lactococcus}$) to 0.058 ($\lambda_{F:G,Enterococcus} / \lambda_{F:G,Staphylococcus}$) for F:G. The complete information for all heritable genera is in Additional file 1: Table S1, Additional file 2: Table S2, Additional file 3: Table S3, and Additional file 4: Table S4.

**Table 2:** Estimates of genetic ($r_{g}$) and phenotypic ($r_{p}$) correlations and of regression coefficients $\lambda_{i,Genus}$ between the considered genera and each of the four traits

Trait	Genus^a^	$r_{g}$	SE	$r_{p}$	SE	$\lambda_{i,Genus}$^b^	SE
P utilization	*Bacillus*	0.36	0.35	0.14	0.04	0.081	0.026
	*Enterococcus*	− 0.19	0.38	− 0.09	0.04	− 0.039	0.016
	*Escherichia*/*Shigella*	− 0.07	0.35	− 0.07	0.04	− 0.026	0.014
	*Lactococcus*	0.52	0.36	0.13	0.04	0.055	0.020
	*Leuconostoc*	0.48	0.35	0.13	0.04	0.070	0.024
	*Streptococcus*	− 0.14	0.37	− 0.09	0.04	− 0.030	0.013
Feed intake	*Bacillus*	0.35	0.34	0.29	0.06	0.095	0.023
	*Curtobacterium*	0.17	0.36	0.20	0.05	0.029	0.009
	*Lactococcus*	0.37	0.37	0.27	0.06	0.057	0.018
	*Leuconostoc*	0.47	0.34	0.31	0.07	0.082	0.021
	*Microbacterium*	− 0.03	0.40	0.17	0.05	0.028	0.011
Body weight gain	*Bacillus*	0.35	0.39	0.23	0.05	0.102	0.025
	*Curtobacterium*	0.41	0.37	0.19	0.04	0.035	0.010
	*Enterococcus*	− 0.69	0.33	− 0.15	0.04	− 0.054	0.015
	*Lactococcus*	0.48	0.40	0.24	0.05	0.076	0.019
	*Leuconostoc*	0.47	0.39	0.25	0.05	0.097	0.023
	*Macrococcus*	0.15	0.37	− 0.16	0.07	− 0.024	0.012
	*Streptococcus*	0.16	0.42	− 0.11	0.04	− 0.056	0.013
Feed per gain	*Aerococcus*	0.25	0.41	0.12	0.04	0.038	0.011
	*Clostridium *sensu stricto	0.15	0.37	− 0.11	0.04	− 0.050	0.013
	*Cutibacterium*	− 0.25	0.41	0.09	0.04	0.027	0.011
	*Enterococcus*	0.54	0.40	0.17	0.04	0.058	0.016
	*Escherichia*/*Shigella*	0.01	0.41	− 0.09	0.04	− 0.035	0.014
	*Lactococcus*	− 0.38	0.43	− 0.07	0.05	− 0.064	0.020
	*Ruminococcus 2*	− 0.33	0.47	0.11	0.04	0.039	0.015
	*Sellimonas*	− 0.27	0.48	0.10	0.04	0.014	0.006
	*Staphylococcus*	− 0.13	0.43	0.14	0.04	0.058	0.020
	*Streptococcus*	0.32	0.43	0.12	0.04	0.038	0.013

*SE* standard errors

^a^All genera with a significant heritability (p ≤ 0.05), significant Pearson correlation (p ≤ 0.05) and significant structural coefficient (p ≤ 0.05) between the genus and the considered trait

^b^In units $$\sigma_{p}$$

### QTL mapping results

**Fig. 1:**
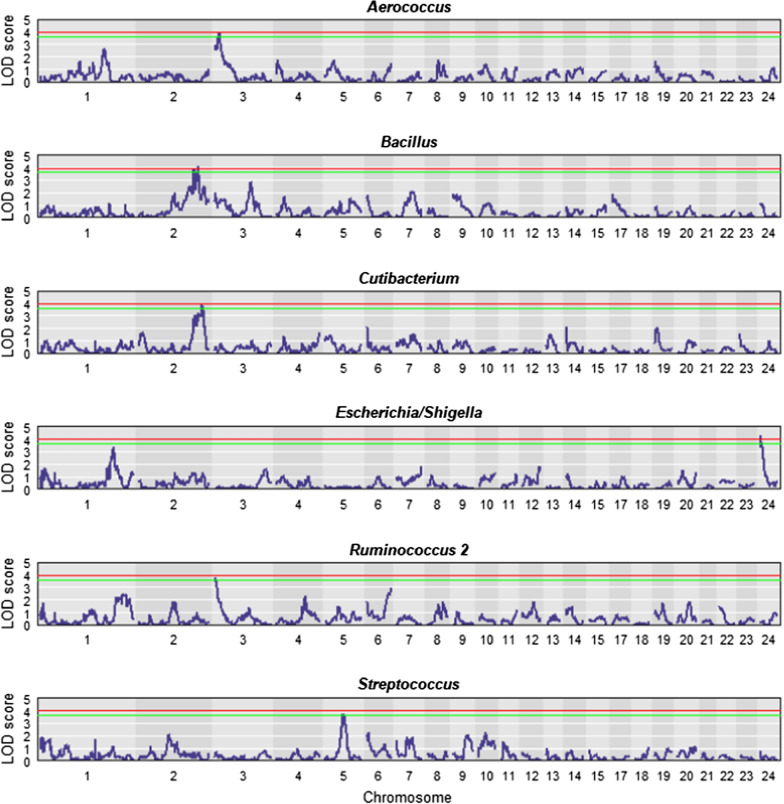
Plots of the QTL linkage mapping scan of heritable genera with significant QTL. QTL linkage mapping scan plots of heritable genera (p value ≤ 0.05) with significant QTL. The LOD score is the test statistic, and the red and green lines correspond to genome-wide significance levels of 5 and 10%, respectively

The QTL linkage mapping results are presented as genome scan plots for each heritable microbial genus with significant QTL in Fig. 1. For clarity, only the first 23 *Coturnix japonica* chromosomes (CJA) are shown within the plots, since no significant peaks were observed for the other chromosomes. As described in Vollmar et al. [18], none of the genotyped SNPs were located on CJA16. Six QTL with genome-wide significance thresholds of 5 and 10% were found across all genera (Table 3). Significant peaks were detected for the microbial genera *Aerococcus* on CJA3, for *Bacillus* on CJA2, for *Cutibacterium* on CJA2, for *Escherichia/Shigella* on CJA24, for *Ruminococcus 2* on CJA3, and for *Streptococcus* on CJA5.

**Table 3:** Results of the QTL linkage mapping

Trait	CJA	Pos (cM)	LOD	Support interval borders (cM)		Number of significant SNPs
				Low	High	
*Aerococcus*	3	12	4.00**	0	19	16
*Bacillus*	2	160	4.14**	147	164	24
*Cutibacterium*	2	171	3.83*	147	178	34
*Escherichia/Shigella*	24	0	4.31**	0	8	2
*Ruminococcus 2*	3	2	3.74*	0	10	10
*Streptococcus*	5	50	3.78*	44	57	17

Pos: positions in cM of 5% (**) and 10% (*) genome-wide significant QTL on *Coturnix japonica* chromosomes (CJA), with LOD score test statistics (LOD) and the corresponding QTL support intervals (SI), in cM. SI_low and SI_high represent the beginning and the end of the SI, respectively, and significant SNPs (p ≤ 0.05) are obtained from the SNP-trait association analysis. The corresponding genetic linkage map can be found in Vollmar et al. [18]

Results of the SNP-based association analyses for SNPs within the SI regions of the six identified QTL are in Table 3. Significant SNPs were found for each SI region, for a total of 103 significant SNPs, as listed in Additional file 5: Table S5. Due to overlapping SI, significant SNPs were shared between *Bacillus* and *Cutibacterium* on CJA2 and between *Aerococcus* and *Ruminococcus 2* on CJA3 (see Additional file 5: Table S5).

### Genomic and microbial trait predictions

Estimates of heritability for the animal microbiota effects for the four traits based on Model (6) were 0.07 (SE = 0.04, p value = 0.020) for PU, 0.14 (SE = 0.05, p value ≤ 0.001) for FI, 0.06 (SE = 0.04, p value = 0.020) for BWG, and 0.03 (SE = 0.03, p value = 0.267) for F:G. For all traits, except F:G, the estimate of heritability of animal microbiota effects was significant (p ≤ 0.05). The results of the cross-validation of microbial and genomic predictions are in Table 4. Genomic predictions, ${\hat{\mathbf{g}}}$, and genomic predictions of the microbiota-mediated part of the traits, ${\hat{\mathbf{m}}}$, had similar correlations with the trait phenotypes. Average correlations between the microbial predictions, ${\hat{\mathbf{k}}}$, and the trait phenotypes were slightly higher than GBLUP accuracies for PU and FI, and markedly higher for BWG and F:G.

**Table 4:** Mean accuracy and confidence interval (CI) of the genomic and microbial trait predictions

Trait	MBLUP		GBLUP		Microbiota-mediated GBLUP	
	Accuracy	95% CI	Accuracy	95% CI	Accuracy	95% CI
PU	0.22	0.09:0.35	0.18	0.05:0.32	0.16	0.01:0.31
FI	0.31	0.17:0.43	0.24	0.10:0.35	0.22	0.07:0.38
BWG	0.34	0.20:0.46	0.13	− 0.01:0.25	0.14	− 0.03:0.29
F:G	0.31	0.10:0.47	0.10	− 0.05:0.23	0.07	− 0.07:0.23

Estimated accuracy of the MBLUP and GBLUP of the trait observations and GBLUP of the microbiota-mediated part of the trait observations

*PU* P utilization, *FI* feed intake, *BWG* body weight gain, *F:G* feed per gain

## Discussion

In previous studies, we investigated the impact of host genetics [15, 18, 23] and of the composition of the ileum microbiota [16, 17] on PU and related traits in Japanese quail. To complement these studies, here we modeled the microbiota composition as a host trait and investigated how the microbiota composition and the host genome can predict the four host phenotypic traits considered in this study.

It is well known that gut microbial colonization is determined by the environmental and genetic background of animals. External factors, such as diet, husbandry, photoperiod, and litter can overlay or mask the effects of host genetics [38–41]. To reduce external influences on gut microbiota and to ensure comparability of animals, standardized housing and management conditions were used for all animals in this study. The microbiota composition DNA samples used in this study originated from an experiment that took place several years ago [15], and at that time, the importance of having control samples of feed, water, litter, DNA extraction, etc. was underestimated.

A large fraction of the bacterial genera showed a significant estimate of heritability of their relative abundance (Table 1). The three genera with the highest heritability estimates were *Clostridium *sensu stricto (0.17), *Lactobacillus* (0.12), and *Bifidobacterium* (0.10). These heritability estimates are lower than those calculated by Camarinha-Silva et al. [10] and Estellé et al. [42] for ileal bacterial genera in pigs and by Org et al. [43] in mice, but a solid comparison across species is questionable. Mignon-Grasteau et al. [13] estimated moderate heritabilities for relative abundance of members of the genera *Lactobacillus* and *Clostridium* in the ceca of chickens.

To examine the relationship between the quantitative traits and composition of the ileal microbiota, we applied SEM, which separate the direct and indirect genetic relationships between the considered traits [44]. Direct genetic effects are due to pleiotropic QTL or linkage disequilibrium between QTL [44]. In the present study, indirect genetic effects were due to the recursive effect of bacterial genera on the host phenotype traits. The selected equation models were modeled in a simplified way and no competing models were compared, in part because the size of the data set was too limited to do this in a thorough manner. It has to be acknowledged that the assumption that the microbiota affects host phenotype traits is questionable, especially for FI, and, therefore, these results have to be interpreted with some caution. Interactions between microbiota and quantitative traits by using structural equation modeling have rarely been studied, except by Saborío-Montero et al. [8] for methane emission in cattle and by Tiezzi et al. [20] for fat deposition in pigs. Nonzero estimates for $\lambda_{PU,Genus}$, $\lambda_{FI,Genus}$, $\lambda_{BWG,Genus}$, and $\lambda_{F:G,Genus}$ (Table 2) indicate that there is a functional link between the four traits and some microbial genera, and the small standard errors confirm the correct directional assumption of the recursive relationship between the bacterial genera and the performance traits.

The main genera that were found to be involved in the recursive relationships with the host traits were *Bacillus*, *Lactococcus* and *Leuconostoc*, albeit with weak signals. Negative recursive interactions with PU were shown for the three genera *Enterococcus*, *Escherichia/Shigella* and *Streptococcus*, and positive recursive interactions were shown between these three same genera and F:G (Table 2). A negative interaction between *Enterococcus* and BWG was also found and a joint positive recursive effect was identified for *Curtobacterium* with both FI and BWG. For F:G, the negative interaction with *Escherichia/Shigella* and *Lactococcus* is worth mentioning, as well as the positive interaction with *Enterococcus,* and *Streptococcus* (Table 2). Our results are in agreement with those of Vollmar et al. [17], who reported an association of *Bacillus* and *Leuconostoc* with PU using a MWAS, and also with those of Borda-Molina et al. [16], who confirmed a positive phenotypic association of the relative abundance of *Bacillus* and *Leuconostoc* in the ileum with PU. Lactic acid bacteria are known to be phytase degraders and some species of the genus *Bacillus* showed extracellular phytase activity that might improve PU efficiency [45, 46].

The phenotypic correlations between the bacterial genera and each of the four performance traits were within a low to medium range (Table 2). Because of the limited number of animals in our study, estimates of genetic correlations (Table 2) had large standard errors and they should be viewed as a supplement to the main results of our study.

To date, only a few QTL studies have been conducted in quail [18, 47–49], with QTL mapped for different behavioral and performance traits. However, several authors have investigated host QTL for microbial colonization of the GIT in other species. For instance, Mignon-Grasteau et al. [13] performed QTL analyses of microbial genera in the ceca of chickens. In our study, six significant host QTL for microbial composition in quail were detected (Fig. 1). One of these significant QTL was for *Bacillus*, on CJA2. Relative abundance of *Bacillus* was most highly correlated and showed the highest recursive relationships with PU, FI, and BWG. Interestingly, the SI of a previously identified QTL for growth rate on this chromosome [48] overlaps with the SI of the QTL on CJA2 for *Bacillus*. Similarly, Essa et al. [50] identified a QTL for BWG on chicken chromosome 2. The SI for the QTL on CJA2 for *Bacillus* also overlapped with that of the QTL for *Cutibacterium* (Table 3) and several common significant SNPs were detected (see Additional file 5: Table S5). The SI of the QTL identified for *Aerococcus* on CJA3 overlapped with that of *Ruminococcus 2*, and several common SNPs were also detected for this overlapping region. On CJA5, the SI of a QTL for *Streptococcus* overlapped with the SI for QTL for FI and foot ash reported in Vollmar et al. [18].

All microbial genera for which significant QTL were mapped were also significantly correlated or significantly linked via recursive interaction with at least one of the host traits, as described above. Thus, it can be assumed that the QTL detected on CJA2 for *Bacillus* directly influences the microbial colonization of the ileum with *Bacillus* and indirectly influences PU, FI, and BWG. The use of SEM for QTL mapping may also be of interest for future studies [51].

Several studies have revealed possible mechanisms for the positive or negative effects of bacterial genera on quantitative traits. Among the bacterial genera with the highest effects on the four traits analyzed here, the genus *Bacillus* was most important. It is used as a probiotic in chickens and improves performance traits [52, 53], positively affects the immune system [54–56], increases digestive enzyme activity [55–57], and synthesizes phytases [58]. A previous study has reported that some strains of *Leuconostoc* have weak enzymatic activities, including the formation of acid phosphatase [59]. In addition, an immunomodulatory activity due to induced cytokine production has been reported in chicken [60]. Some subspecies of *Lactococcus* supplied in the diet of broilers have resulted in a lower F:G, increased body weight, and reduced mortality, with positive effects on the immune system [61, 62] and carcass quality [63]. As noted above, relative abundance of the genus *Enterococcus* has a negative impact on PU, FI and BWG. In humans, some members of this genus are considered to be opportunistic pathogens due to their antibiotic resistance [64, 65]. In chickens, these bacteria can lead to increased one-day mortality [66], and the formation of toxic metabolites by bacterial metabolization of protein has also been reported [67]. Relative abundance of the genera *Staphylococcus* and *Streptococcus* also negatively influenced the traits analyzed here, and have been shown to cause different diseases and affect poultry health with, depending on the bacterial species, several clinical observations that range from drowsiness and poor feed intake to increased mortality (reviewed in [68]).

Results from the microbial and genomic predictions (Table 4) confirmed the strong impact of the host genome and microbiota composition on the analyzed host traits. The two-step procedure proposed by Weishaar et al. [21] to estimate breeding values for the microbiota-mediated part of a trait was also successful in our study, in particular for PU and FI. The GBLUP accuracy for the microbiota-mediated part of the host phenotype was only slightly lower than the prediction accuracy of the conventional GBLUP. One explanation for this result might be that the genetic effect of the host on the trait mediated by the microbiota is much stronger than the direct genetic effect of the host. To substantiate this hypothesis, we fitted Model (1) with an additional random animal effect with the microbiota-based covariance matrix ${\mathbf{M}}$ for PU. Compared to a model with only the microbiota effect, the estimate of microbiability for PU remained at almost the same level (0.15) but the estimate of heritability dropped from 0.12 to 0.07 (results not shown). A similar pattern was observed by Difford et al. [9] in a study on dairy cattle rumen microbiota composition and methane production. This clearly shows that fitting both random effects simultaneous is beneficial but that assuming a zero covariance between the two random effects is too simplistic. How to model both effects simultaneously and how to interpret the results from such models biologically is an ongoing research topic [69–71] but this is outside the scope of our study.

## Conclusions

We detected a significant genetic effect of the host on composition of the ileum microbiota in quail. Among the 59 bacterial genera, 24 showed a significant heritability of their relative abundance. The estimated correlations of the bacterial genera with the four host traits analyzed (PU, FI, BWG, and F:G) and the calculated recursive effects from the SEM confirmed the recursive relationship between the relative abundance of individual bacterial genera and these traits. Several significant QTL were identified for microbiota composition, which were supported by trait-associated SNPs (associated with PU, FI, BWG, or F:G). The application of microbial and genomic mixed linear models allowed accurate prediction of PU and the related traits. In particular, applying these models made it possible to predict the microbiota-mediated part of the traits, demonstrating the feasibility of hologenomic selection.

## Supplementary Information

**Additional file 1: Table S1.** Genetic correlations $r_{g}$, phenotypic correlations $r_{p}$ and regression coefficients $\lambda_{PU, Genus}$*.* Correlations and regression coefficients between PU and Genus with significant heritability (p ≤ 0.05) The standard errors (SE) presented in parantheses and $\lambda_{PU, Genus}$ in units $\sigma_{p}$. ^1^P utilization—Genus with significant heritability (p ≤ 0.05).

**Additional file 2: Table S2.** Genetic correlations $r_{g}$, phenotypic correlations $r_{p}$ and regression coefficients $\lambda_{{FI,{ }Genus}}$. Correlations and regression coefficients between FI and Genus with significant heritability (p ≤ 0.05) The standard errors (SE) presented in parantheses and $\lambda_{FI, Genus}$ in units $\sigma_{p}$. ^1^Feed intake—Genus with significant heritability (p ≤ 0.05).

**Additional file 3: Table S3.** Genetic correlations $r_{g}$, phenotypic correlations $r_{p}$ and regression coefficients $\lambda_{BWG, Genus}$. Correlations and regression coefficients between BWG and Genus with significant heritability (p ≤ 0.05) The standard errors (SE) presented in parantheses and $\lambda_{BWG, Genus}$ in units $\sigma_{p}$. ^1^Body weight gain—Genus with significant heritability (p ≤ 0.05).

**Additional file 4: Table S4.** Genetic correlations $r_{g}$, phenotypic correlations $r_{p}$ and regression coefficients $\lambda_{F:G, Genus}$. Correlations and regression coefficients between F:G and Genus with significant heritability (p ≤ 0.05) The standard errors (SE) presented in parantheses and $\lambda_{F:G, Genus}$ in units $\sigma_{p}$. ^1^Feed per gain—Genus with significant heritability (p ≤ 0.05).

**Additional file 5: Table S5.** Trait-associated markers from GCTA within the significant QTL regions. Summary of the trait-associated markers from GCTA (p ≤ 0.05) within the significant QTL regions. In addition, all markers that are significantly associated with another characteristic are listed in the last column. ^1^in cM.

## Data Availability

Not applicable.
