# Supplementary material for: Composition of the ileum microbiota is a mediator between the host genome and phosphorus utilization and other efficiency traits in Japanese quail (Coturnix japonica)
Source: Genet Sel Evol. 2022 Mar 8;54:20. doi: 10.1186/s12711-022-00697-8 (PMC8903610; doi:10.1186/s12711-022-00697-8)
Supplement: Supplementary file 1 — Additional file 1: Table S1. Genetic correlations $r_{g}$rg, phenotypic correlations $r_{p}$rp and regression coefficients $\lambda_{PU, Genus}$λPU,Genus. Correlations and regression coefficients between PU and Genus with significant heritability (p ≤ 0.05) The standard errors (SE) presented in parantheses and $\lambda_{PU, Genus}$λPU,Genus in units $\sigma_{p}$σp. 1P utilization—Genus with significant heritability (p ≤ 0.05). [file 12711_2022_697_MOESM1_ESM.docx]

**Additional file 1: Table S1** **Correlations and regression coefficients between PU and Genera with significant heritability.**

| **Traits**^1^ | $\boldsymbol{r}_{\boldsymbol{g}}$ | **(SE)** | $\boldsymbol{r}_{\boldsymbol{p}}$ | **(SE)** | $\boldsymbol{\lambda}_{\boldsymbol{PU, Genus}}$ | **(SE)** |
| --- | --- | --- | --- | --- | --- | --- |
| PU - *Aerococcus* | -0.628 | (0.307) | -0.021 | (0.039) | 0.019 | (0.011) |
| PU - *Anaerostipes* | 0.312 | (0.397) | 0.075 | (0.040) | 0.008 | (0.005) |
| PU - *Bacillus* | 0.357 | (0.349) | 0.141 | (0.040) | 0.081 | (0.026) |
| PU - *Bifidobacterium* | 0.191 | (0.320) | -0.002 | (0.042) | -0.003 | (0.017) |
| PU - *Clostridium* *sensu* *stricto* | -0.283 | (0.315) | -0.025 | (0.040) | 0.009 | (0.013) |
| PU - *Corynebacterium* | -0.660 | (0.389) | 0.003 | (0.039) | 0.021 | (0.010) |
| PU - *Corynebacterium* | -0.490 | (0.357) | -0.012 | (0.041) | 0.023 | (0.014) |
| PU - *Curtobacterium* | 0.188 | (0.382) | 0.074 | (0.038) | 0.017 | (0.010) |
| PU - *Cutibacterium* | 0.222 | (0.356) | -0.001 | (0.040) | -0.006 | (0.011) |
| PU - *Enterococcus* | -0.192 | (0.380) | -0.085 | (0.038) | -0.039 | (0.016) |
| PU - *Escherichia*/*Shigella* | -0.065 | (0.347) | -0.065 | (0.040) | -0.026 | (0.014) |
| PU - *Lactobacillus* | 0.112 | (0.329) | -0.038 | (0.047) | -0.009 | (0.010) |
| PU - *Lactococcus* | 0.523 | (0.355) | 0.129 | (0.041) | 0.055 | (0.020) |
| PU - *Leuconostoc* | 0.480 | (0.349) | 0.134 | (0.042) | 0.070 | (0.024) |
| PU - *Macrococcus* | -0.130 | (0.336) | -0.006 | (0.053) | 0.021 | (0.012) |
| PU - *Microbacterium* | -0.022 | (0.401) | 0.042 | (0.039) | 0.018 | (0.012) |
| PU - *Ruminococcus* *2* | 0.462 | (0.359) | 0.027 | (0.040) | 0.007 | (0.015) |
| PU - *Sellimonas* | 0.416 | (0.368) | 0.056 | (0.039) | 0.005 | (0.006) |
| PU - *Staphylococcus* | 0.839 | (0.264) | 0.019 | (0.045) | -0.029 | (0.020) |
| PU - *Streptococcus* | -0.143 | (0.366) | -0.086 | (0.038) | -0.030 | (0.013) |
| PU - *Subdoligranulum* | 0.534 | (0.354) | 0.052 | (0.039) | 0.005 | (0.006) |
| PU - *Tyzzerella* | -0.384 | (0.378) | 0.018 | (0.040) | < 0.001 | (< 0.001) |
| PU - Unc. *Lachnospiraceae* | 0.292 | (0.364) | 0.053 | (0.040) | 0.022 | (0.014) |

Genetic correlations $r_{g}$, phenotypic correlations $r_{p}$ and regression coefficients $\lambda_{PU, Genus}$ between PU and Genera with significant heritability (p ≤ 0.05). The standard errors (SE) presented in parantheses and $\lambda_{PU, Genus}$ in units $\sigma_{p}$. ^1^ P utilization – Genus with significant heritability (p ≤ 0.05).
