# Supplementary material for: Composition of the ileum microbiota is a mediator between the host genome and phosphorus utilization and other efficiency traits in Japanese quail (Coturnix japonica)
Source: Genet Sel Evol. 2022 Mar 8;54:20. doi: 10.1186/s12711-022-00697-8 (PMC8903610; doi:10.1186/s12711-022-00697-8)
Supplement: Supplementary file 2 — Additional file 2: Table S2. Genetic correlations $r_{g}$rg, phenotypic correlations $r_{p}$rp and regression coefficients $\lambda_{{FI,{ }Genus}}$λFI,Genus. Correlations and regression coefficients between FI and Genus with significant heritability (p ≤ 0.05) The standard errors (SE) presented in parantheses and $\lambda_{FI, Genus}$λFI,Genus in units $\sigma_{p}$σp. 1Feed intake—Genus with significant heritability (p ≤ 0.05). [file 12711_2022_697_MOESM2_ESM.docx]

**Additional file 2: Table S2 Correlations and regression coefficients between FI and Genera with significant heritability.**

| **Traits**^1^ | $\boldsymbol{r}_{\boldsymbol{g}}$ | **(SE)** | $\boldsymbol{r}_{\boldsymbol{p}}$ | **(SE)** | $\boldsymbol{\lambda}_{\boldsymbol{FI, Genus}}$ | **(SE)** |
| --- | --- | --- | --- | --- | --- | --- |
| FI - *Aerococcus* | -0.210 | (0.358) | -0.073 | (0.048) | 0.007 | (0.010) |
| FI - *Anaerostipes* | 0.414 | (0.394) | 0.125 | (0.059) | 0.001 | (0.004) |
| FI - *Bacillus* | 0.347 | (0.340) | 0.290 | (0.059) | 0.095 | (0.023) |
| FI - *Bifidobacterium* | 0.093 | (0.323) | 0.138 | (0.061) | 0.021 | (0.015) |
| FI - *Clostridium sensu stricto* | -0.205 | (0.318) | -0.017 | (0.047) | 0.028 | (0.012) |
| FI - *Corynebacterium* | -0.430 | (0.387) | -0.052 | (0.054) | 0.022 | (0.009) |
| FI - *Corynebacterium* | -0.258 | (0.367) | -0.100 | (0.058) | 0.013 | (0.012) |
| FI - *Curtobacterium* | 0.168 | (0.360) | 0.195 | (0.047) | 0.029 | (0.009) |
| FI - *Cutibacterium* | 0.053 | (0.350) | 0.122 | (0.056) | 0.015 | (0.010) |
| FI - *Enterococcus* | -0.576 | (0.310) | -0.077 | (0.041) | -0.022 | (0.014) |
| FI - *Escherichia/Shigella* | -0.380 | (0.334) | -0.061 | (0.053) | 0.006 | (0.012) |
| FI - *Lactobacillus* | -0.097 | (0.314) | -0.202 | (0.084) | 0.001 | (0.010) |
| FI - *Lactococcus* | 0.366 | (0.365) | 0.270 | (0.064) | 0.057 | (0.018) |
| FI - *Leuconostoc* | 0.466 | (0.338) | 0.307 | (0.065) | 0.082 | (0.021) |
| FI - *Macrococcus* | 0.291 | (0.312) | -0.226 | (0.103) | 0.003 | (0.012) |
| FI - *Microbacterium* | -0.026 | (0.395) | 0.165 | (0.051) | 0.028 | (0.011) |
| FI - *Ruminococcus* *2* | 0.425 | (0.396) | 0.084 | (0.054) | -0.002 | (0.013) |
| FI - *Sellimonas* | 0.340 | (0.395) | 0.119 | (0.052) | 0.002 | (0.005) |
| FI - *Staphylococcus* | 0.563 | (0.327) | 0.168 | (0.080) | -0.020 | (0.019) |
| FI - *Streptococcus* | 0.116 | (0.372) | -0.054 | (0.043) | -0.038 | (0.012) |
| FI - *Subdoligranulum* | 0.369 | (0.399) | 0.074 | (0.046) | 0.004 | (0.005) |
| FI - *Tyzzerella* | -0.264 | (0.376) | 0.083 | (0.054) | < 0.001 | (< 0.001) |
| FI - Unc. *Lachnospiraceae* | 0.334 | (0.368) | 0.112 | (0.054) | 0.008 | (0.013) |

Genetic correlations $r_{g}$, phenotypic correlations $r_{p}$ and regression coefficients $\lambda_{FI, Genus}$ between FI and Genera with significant heritability (p ≤ 0.05). The standard errors (SE) presented in parantheses and $\lambda_{FI, Genus}$ in units $\sigma_{p}$. ^1^ Feed intake – Genus with significant heritability (p ≤ 0.05).
