# Supplementary material for: Composition of the ileum microbiota is a mediator between the host genome and phosphorus utilization and other efficiency traits in Japanese quail (Coturnix japonica)
Source: Genet Sel Evol. 2022 Mar 8;54:20. doi: 10.1186/s12711-022-00697-8 (PMC8903610; doi:10.1186/s12711-022-00697-8)
Supplement: Supplementary file 3 — Additional file 3: Table S3. Genetic correlations $r_{g}$rg, phenotypic correlations $r_{p}$rp and regression coefficients $\lambda_{BWG, Genus}$λBWG,Genus. Correlations and regression coefficients between BWG and Genus with significant heritability (p ≤ 0.05) The standard errors (SE) presented in parantheses and $\lambda_{BWG, Genus}$λBWG,Genus in units $\sigma_{p}$σp. 1Body weight gain—Genus with significant heritability (p ≤ 0.05). [file 12711_2022_697_MOESM3_ESM.docx]

**Additional file 3: Table S3 Correlations and regression coefficients between BWG and Genera with significant heritability.**

| **Traits**^1^ | $\boldsymbol{r}_{\boldsymbol{g}}$ | **(SE)** | $\boldsymbol{r}_{\boldsymbol{p}}$ | **(SE)** | $\boldsymbol{\lambda}_{\boldsymbol{BWG, Genus}}$ | **(SE)** |
| --- | --- | --- | --- | --- | --- | --- |
| BWG - *Anaerofilum* | 0.202 | (0.464) | -0.044 | (0.040) | -0.009 | (0.001) |
| BWG - *Anaerostipes* | 0.319 | (0.472) | 0.057 | (0.047) | < 0.001 | (0.005) |
| BWG - *Bacillus* | 0.351 | (0.392) | 0.232 | (0.048) | 0.102 | (0.025) |
| BWG - *Bifidobacterium* | 0.116 | (0.364) | 0.056 | (0.050) | -0.001 | (0.017) |
| BWG - *Clostridium sensu stricto* | -0.385 | (0.355) | 0.044 | (0.042) | 0.051 | (0.013) |
| BWG - *Corynebacterium* | -0.444 | (0.435) | -0.048 | (0.045) | 0.010 | (0.010) |
| BWG - *Corynebacterium* | -0.160 | (0.415) | -0.110 | (0.047) | -0.013 | (0.013) |
| BWG - *Curtobacterium* | 0.413 | (0.374) | 0.185 | (0.041) | 0.035 | (0.010) |
| BWG - *Cutibacterium* | 0.143 | (0.394) | 0.043 | (0.047) | -0.003 | (0.011) |
| BWG - *Enterococcus* | -0.685 | (0.332) | -0.149 | (0.038) | -0.054 | (0.015) |
| BWG - *Escherichia/Shigella* | -0.583 | (0.379) | 0.007 | (0.045) | 0.033 | (0.013) |
| BWG - *Lactobacillus* | 0.213 | (0.355) | -0.092 | (0.064) | -0.002 | (0.010) |
| BWG - *Lactococcus* | 0.479 | (0.399) | 0.236 | (0.050) | 0.076 | (0.019) |
| BWG - *Leuconostoc* | 0.471 | (0.392) | 0.248 | (0.051) | 0.097 | (0.023) |
| BWG - *Macrococcus* | 0.150 | (0.371) | -0.161 | (0.072) | -0.024 | (0.012) |
| BWG - *Microbacterium* | 0.292 | (0.427) | 0.115 | (0.044) | 0.015 | (0.012) |
| BWG - *Ruminococcus* *2* | 0.455 | (0.460) | -0.001 | (0.045) | -0.021 | (0.014) |
| BWG - *Sellimonas* | 0.342 | (0.454) | 0.033 | (0.045) | -0.005 | (0.006) |
| BWG - *Staphylococcus* | 0.458 | (0.395) | 0.045 | (0.062) | -0.042 | (0.020) |
| BWG - *Streptococcus* | 0.157 | (0.416) | -0.109 | (0.039) | -0.056 | (0.013) |
| BWG - *Subdoligranulum* | 0.449 | (0.471) | 0.003 | (0.041) | -0.005 | (0.005) |
| BWG - *Tyzzerella* | -0.312 | (0.414) | 0.071 | (0.045) | < 0.001 | (< 0.001) |
| BWG - Unc. *Lachnospiraceae* | 0.358 | (0.426) | 0.020 | (0.045) | -0.014 | (0.014) |

Genetic correlations $r_{g}$, phenotypic correlations $r_{p}$ and regression coefficients $\lambda_{BWG, Genus}$ between BWG and Genera with significant heritability (p ≤ 0.05). The standard errors (SE) presented in parantheses and $\lambda_{BWG, Genus}$ in units $\sigma_{p}$. ^1^ Body weight gain – Genus with significant heritability (p ≤ 0.05).
