# Supplementary material for: Composition of the ileum microbiota is a mediator between the host genome and phosphorus utilization and other efficiency traits in Japanese quail (Coturnix japonica)
Source: Genet Sel Evol. 2022 Mar 8;54:20. doi: 10.1186/s12711-022-00697-8 (PMC8903610; doi:10.1186/s12711-022-00697-8)
Supplement: Supplementary file 4 — Additional file 4: Table S4. Genetic correlations $r_{g}$rg, phenotypic correlations $r_{p}$rp and regression coefficients $\lambda_{F:G, Genus}$λF:G,Genus. Correlations and regression coefficients between F:G and Genus with significant heritability (p ≤ 0.05) The standard errors (SE) presented in parantheses and $\lambda_{F:G, Genus}$λF:G,Genus in units $\sigma_{p}$σp. 1Feed per gain—Genus with significant heritability (p ≤ 0.05). [file 12711_2022_697_MOESM4_ESM.docx]

**Additional file 4: Table S4 Correlations and regression coefficients between F:G and Genera with significant heritability.**

| **Traits**^1^ | $\boldsymbol{r}_{\boldsymbol{g}}$ | **SE** | $\boldsymbol{r}_{\boldsymbol{p}}$ | **SE** | $\boldsymbol{\lambda}_{\boldsymbol{F:G, Genus}}$ | **SE** |
| --- | --- | --- | --- | --- | --- | --- |
| F:G - *Aerococcus* | 0.247 | (0.408) | 0.120 | (0.038) | 0.038 | (0.011) |
| F:G - *Anaerofilum* | -0.410 | (0.453) | 0.043 | (0.038) | 0.002 | (0.001) |
| F:G - *Bacillus* | -0.224 | (0.424) | -0.052 | (0.043) | -0.068 | (0.026) |
| F:G - *Bifidobacterium* | -0.084 | (0.379) | 0.081 | (0.041) | 0.030 | (0.017) |
| F:G - *Clostridium sensu stricto* | 0.151 | (0.368) | -0.107 | (0.038) | -0.050 | (0.013) |
| F:G - *Corynebacterium* | 0.300 | (0.475) | 0.024 | (0.039) | 0.008 | (0.010) |
| F:G - *Corynebacterium* | -0.018 | (0.445) | 0.053 | (0.041) | 0.033 | (0.014) |
| F:G - *Curtobacterium* | -0.578 | (0.370) | -0.065 | (0.039) | -0.021 | (0.010) |
| F:G - *Cutibacterium* | -0.254 | (0.407) | 0.089 | (0.040) | 0.027 | (0.011) |
| F:G - *Enterococcus* | 0.539 | (0.397) | 0.165 | (0.036) | 0.058 | (0.016) |
| F:G - *Escherichia/Shigella* | 0.012 | (0.405) | -0.086 | (0.039) | -0.035 | (0.014) |
| F:G - *Lactobacillus* | -0.521 | (0.326) | -0.087 | (0.047) | 0.013 | (0.010) |
| F:G - *Lactococcus* | -0.383 | (0.431) | -0.067 | (0.045) | -0.064 | (0.020) |
| F:G - *Leuconostoc* | -0.201 | (0.448) | -0.054 | (0.046) | -0.080 | (0.023) |
| F:G - *Macrococcus* | 0.246 | (0.381) | -0.004 | (0.060) | 0.047 | (0.011) |
| F:G - *Microbacterium* | -0.595 | (0.395) | 0.001 | (0.039) | 0.006 | (0.013) |
| F:G - *Ruminococcus* *2* | -0.334 | (0.466) | 0.106 | (0.039) | 0.039 | (0.015) |
| F:G - *Sellimonas* | -0.273 | (0.476) | 0.102 | (0.039) | 0.014 | (0.006) |
| F:G - *Staphylococcus* | -0.125 | (0.432) | 0.137 | (0.043) | 0.058 | (0.020) |
| F:G - *Streptococcus* | 0.321 | (0.425) | 0.118 | (0.037) | 0.038 | (0.013) |
| F:G - *Tyzzerella* | 0.149 | (0.435) | -0.011 | (0.040) | < 0.001 | (< 0.001) |
| F:G - Unc. *Lachnospiraceae* | -0.247 | (0.448) | 0.099 | (0.039) | -0.002 | (0.014) |

Genetic correlations $r_{g}$, phenotypic correlations $r_{p}$ and regression coefficients $\lambda_{F:G, Genus}$ between F:G and Genera with significant heritability (p ≤ 0.05). The standard errors (SE) presented in parantheses and $\lambda_{F:G, Genus}$ in units $\sigma_{p}$. ^1^ Feed per gain – Genus with significant heritability (p ≤ 0.05).
