# Supplementary material for: Composition of the ileum microbiota is a mediator between the host genome and phosphorus utilization and other efficiency traits in Japanese quail (Coturnix japonica)
Source: Genet Sel Evol. 2022 Mar 8;54:20. doi: 10.1186/s12711-022-00697-8 (PMC8903610; doi:10.1186/s12711-022-00697-8)
Supplement: Supplementary file 5 — Additional file 5: Table S5. Trait-associated markers from GCTA within the significant QTL regions. Summary of the trait-associated markers from GCTA (p ≤ 0.05) within the significant QTL regions. In addition, all markers that are significantly associated with another characteristic are listed in the last column. 1in cM. [file 12711_2022_697_MOESM5_ESM.docx]

**Additional file 5: Table S5** **Trait-associated markers from GCTA within the significant QTL regions.**

| **Trait** | **CJA** | **Marker ID** | **p value** | **Pos^1^** | **Markers matched with other traits** |
| --- | --- | --- | --- | --- | --- |
| *Aerococcus* | 3 | id00986 | < 0.001 | 0.000 | *Ruminococcus 2* |
|  | 3 | id29156 | 0.027 | 0.000 |  |
|  | 3 | id00575 | < 0.001 | 0.000 | *Ruminococcus 2* |
|  | 3 | id13672 | 0.007 | 0.360 | *Ruminococcus 2* |
|  | 3 | id15191 | 0.028 | 1.830 |  |
|  | 3 | id07523 | 0.016 | 3.082 | *Ruminococcus 2* |
|  | 3 | id10549 | 0.033 | 3.082 |  |
|  | 3 | id02839 | 0.030 | 3.082 | *Ruminococcus 2* |
|  | 3 | id14154 | 0.028 | 8.013 | *Ruminococcus 2* |
|  | 3 | id12388 | 0.019 | 8.024 | *Ruminococcus 2* |
|  | 3 | id24815 | 0.003 | 9.433 |  |
|  | 3 | id18519 | 0.009 | 11.689 |  |
|  | 3 | id03758 | 0.021 | 16.649 |  |
|  | 3 | id13768 | 0.014 | 17.290 |  |
|  | 3 | id05996 | 0.008 | 17.290 |  |
|  | 3 | id12382 | 0.008 | 17.290 |  |
| *Bacillus* | 2 | id09016 | 0.003 | 147.885 |  |
|  | 2 | id05956 | 0.023 | 148.328 |  |
|  | 2 | id32858 | 0.006 | 148.948 |  |
|  | 2 | id12291 | 0.013 | 150.053 |  |
|  | 2 | id33001 | 0.014 | 150.574 | *Cutibacterium* |
|  | 2 | id25447 | 0.008 | 150.911 |  |
|  | 2 | id30476 | 0.021 | 151.894 |  |
|  | 2 | id03102 | 0.002 | 154.051 |  |
|  | 2 | id00923 | 0.003 | 154.051 |  |
|  | 2 | id04053 | 0.002 | 157.198 | *Cutibacterium* |
|  | 2 | id33819 | 0.008 | 157.198 |  |
|  | 2 | id10445 | 0.002 | 157.198 | *Cutibacterium* |
|  | 2 | id13471 | 0.001 | 158.582 |  |
|  | 2 | id06720 | 0.001 | 158.587 | *Cutibacterium* |
|  | 2 | id02198 | < 0.001 | 158.750 | *Cutibacterium* |
|  | 2 | id04692 | 0.041 | 159.867 |  |
|  | 2 | id01497 | 0.002 | 160.198 | *Cutibacterium* |
|  | 2 | id10454 | < 0.001 | 160.198 | *Cutibacterium* |
|  | 2 | id02833 | 0.003 | 160.198 | *Cutibacterium* |
|  | 2 | id02131 | 0.002 | 160.317 | *Cutibacterium* |
|  | 2 | id12311 | 0.028 | 161.185 |  |
|  | 2 | id08123 | 0.028 | 161.185 |  |
|  | 2 | id09029 | 0.011 | 163.378 |  |
|  | 2 | id09032 | 0.032 | 163.648 | *Cutibacterium* |
| *Cutibacterium* | 2 | id33001 | 0.032 | 150.574 | *Bacillus* |
|  | 2 | id04053 | 0.015 | 157.198 | *Bacillus* |
|  | 2 | id10445 | 0.049 | 157.198 | *Bacillus* |
|  | 2 | id18905 | 0.023 | 157.199 |  |
|  | 2 | id06720 | 0.043 | 158.587 | *Bacillus* |
|  | 2 | id02198 | 0.039 | 158.750 | *Bacillus* |
|  | 2 | id01497 | 0.045 | 160.198 | *Bacillus* |
|  | 2 | id10454 | 0.009 | 160.198 | *Bacillus* |
|  | 2 | id02833 | 0.011 | 160.198 | *Bacillus* |
|  | 2 | id02131 | 0.039 | 160.317 | *Bacillus* |
|  | 2 | id09032 | 0.035 | 163.648 | *Bacillus* |
|  | 2 | id01918 | 0.037 | 165.518 |  |
|  | 2 | id13472 | 0.037 | 165.518 |  |
|  | 2 | id32230 | 0.045 | 168.860 |  |
|  | 2 | id15179 | 0.032 | 168.860 |  |
|  | 2 | id32700 | 0.041 | 168.871 |  |
|  | 2 | id17401 | 0.014 | 169.669 |  |
|  | 2 | id23477 | 0.013 | 169.669 |  |
|  | 2 | id28643 | 0.013 | 169.669 |  |
|  | 2 | id08129 | 0.011 | 169.669 |  |
|  | 2 | id10474 | 0.007 | 170.978 |  |
|  | 2 | id31089 | 0.001 | 171.280 |  |
|  | 2 | id12342 | 0.003 | 171.280 |  |
|  | 2 | id08131 | 0.001 | 171.280 |  |
|  | 2 | id15180 | 0.020 | 171.280 |  |
|  | 2 | id03809 | 0.002 | 171.280 |  |
|  | 2 | id01498 | 0.002 | 171.280 |  |
|  | 2 | id09043 | 0.017 | 173.562 |  |
|  | 2 | id32704 | 0.044 | 173.563 |  |
|  | 2 | id10482 | 0.021 | 173.563 |  |
|  | 2 | id17403 | 0.044 | 173.567 |  |
|  | 2 | id13854 | 0.003 | 174.374 |  |
|  | 2 | id00622 | 0.001 | 176.564 |  |
|  | 2 | id17758 | 0.014 | 176.564 |  |
| *Escherichia/Shigella* | 24 | id10195 | 0.042 | 0.000 |  |
|  | 24 | id13533 | 0.041 | 0.000 |  |
| *Ruminococcus 2* | 3 | id00986 | < 0.001 | 0.000 | *Aerococcus* |
|  | 3 | id18286 | 0.039 | 0.000 |  |
|  | 3 | id00575 | 0.001 | 0.000 | *Aerococcus* |
|  | 3 | id13672 | 0.009 | 0.360 | *Aerococcus* |
|  | 3 | id06525 | 0.001 | 0.360 |  |
|  | 3 | id07523 | 0.009 | 3.082 | *Aerococcus* |
|  | 3 | id02839 | 0.014 | 3.082 | *Aerococcus* |
|  | 3 | id34021 | 0.002 | 3.082 |  |
|  | 3 | id14154 | 0.008 | 8.013 | *Aerococcus* |
|  | 3 | id12388 | 0.009 | 8.024 | *Aerococcus* |
| *Streptococcus* | 5 | id14925 | < 0.001 | 44.417 |  |
|  | 5 | id08312 | 0.035 | 44.614 |  |
|  | 5 | id15246 | 0.026 | 45.341 |  |
|  | 5 | id03766 | 0.005 | 45.342 |  |
|  | 5 | id17506 | 0.015 | 45.342 |  |
|  | 5 | id01172 | 0.038 | 45.342 |  |
|  | 5 | id04785 | 0.007 | 45.342 |  |

Summary of the trait-associated markers from GCTA (p ≤ 0.05) within the significant QTL regions. In addition, all markers that are significantly associated with another characteristic are listed in the last column. ^1^ in cM.
